# Assessing the expression of two post-transcriptional BDNF regulators, TTP and miR-16 in the peripheral blood of patients with Schizophrenia

**DOI:** 10.1186/s12888-022-04442-9

**Published:** 2022-12-08

**Authors:** Mohammad Reza Asadi, Jalal Gharesouran, Hani Sabaie, Marziyeh Sadat Moslehian, Hossein Dehghani, Shahram Arsang-Jang, Mohammad Taheri, Deniz Mortazavi, Bashdar Mahmud Hussen, Arezou Sayad, Maryam Rezazadeh

**Affiliations:** 1 Student Research Committee, Tabriz University of Medical Sciences, Tabriz, Iran; 2 Department of Medical Genetics, Faculty of Medicine, Tabriz University of Medical Sciences, Tabriz, Iran; 3 Department of Molecular Medicine, School of Medicine, Birjand University of Medical Sciences, Birjand, Iran; 4 Cancer Gene therapy Research Center, Zanjan University of Medical Science, Zanjan, Iran; 5 Men’s Health and Reproductive Health Research Center, Shahid Beheshti University of Medical Sciences, Tehran, Iran; 6 Department of Genetics and Molecular Biology, School of Medicine, Isfahan university of medical sciences, Isfahan, Iran; 7 Department of Pharmacognosy, College of Pharmacy, Hawler Medical University, Kurdistan Region, Erbil, Iraq; 8 Department of Medical Genetics, School of Medicine, Shahid Beheshti University of Medical Sciences, Tehran, Iran

**Keywords:** Schizophrenia, TTP, miR-16, Expression, BDNF, AU-rich elements, qPCR

## Abstract

Schizophrenia (SCZ) is a severe mental disorder with an unknown pathophysiology. Brain-Derived Neurotrophic Factor (BDNF) is a neurotrophin that has been associated with synapse plasticity, learning, and memory, as well as neurodevelopment and neuroprotection. The importance of neurodevelopmental and neurotoxicity-related components in the pathophysiology of SCZ has been highlighted in research on the neurobiology of this disease. The purpose of this research is to investigate the significant expression of two variables, tristetraprolin (TTP) and miR-16, which are known to be regulators of BDNF expression. Fifty Iranian Azeri SCZ patients were enrolled, and fifty healthy volunteers were age- and gender-matched as controls. A quantitative polymerase chain reaction measured the expression levels of the TTP and miR-16 in the peripheral blood (PB) of SCZ patients and healthy people. TTP expression levels in patients were higher than in controls, regardless of gender or age (posterior beta = 1.532, adjusted *P*-value = 0.012). TTP and miR-16 expression levels were found to be significantly correlated in both SCZ patients and healthy controls (*r* = 0.701, *P* < 0.001 and *r* = 0.777, *P* < 0.001, respectively). Due to the increased expression of TTP in SCZ and the existence of a significant correlation between TTP and miR-16, which helps to act on target mRNAs with AU-rich elements, this mechanism can be considered an influencing factor in SCZ.

## Introduction

Schizophrenia (SCZ) is a mental disorder associated with persistent or recurrent psychosis, affects over 21 million people worldwide, and occurs in late adolescence and early adulthood [1]. Psychosis is an abnormal mental condition in which a person is unable to distinguish between reality and unreality [2]. The main symptoms are summarized as hallucination, delusion, and disorder in behavior, thoughts, and perception [3]. During a standard classification, the symptoms of SCZ fall into two categories: positive and negative. Hallucination, delusion, and formal thought disorders are characterized as positive symptoms, and anhedonia, difficulty in speech, and lack of motivation as negative symptoms [4]. SCZ can be classified as a neurodevelopmental disease in which no precise border can be drawn between the genetic and environmental factors that cause the disease [3]. It is estimated that between 70 and 80% of patients have a risk of developing genetic SCZ [5]. Having a first degree relative to SCZ is the most significant risk factor that increases the incidence rate by 6.5% [6]. When only one parent is affected, the risk is up to 13%, and when both are affected, the risk is up to 50%. Studies on identical twins also show that more than 40% of cases affect both twins [7].

Brain-derived neurotrophic factor (BDNF) is the most significant neurotrophic factor in the central nervous system (CNS) [8]. This condition has been seen in various brain areas, including the hippocampus, cortex, hypothalamus, amygdala, solitary tract nucleus, and substantia nigra. In addition to the CNS, BDNF is also released by peripheral tissues [9]. Animal studies demonstrate that BDNF levels in the brain and serum are positively correlated [10, 11]. BDNF, as an anorexigenic signaling molecule, performs a variety of functions, including neurotrophic activity, energy equilibrium, cardiovascular modulation, food intake, and body weight [12]. Evidence shows that BDNF deficiencies play a role in the etiology of psychiatric diseases such as SCZ due to their fundamental involvement in brain function [13, 14]. Precise BDNF level regulation is crucial in determining the biological result. A 50% reduction in BDNF levels in BDNF knock-out heterozygous mice is related to various CNS abnormalities [15, 16]. In contrast, in a mouse model of Alzheimer’s disease, a 2-fold increase in endogenous BDNF caused by the reduction of microRNA (miR)-206, a direct negative regulator of BDNF levels, alleviates the disease phenotype [17]. Nonetheless, despite their biological and possible clinical importance, the processes that govern BDNF levels are not entirely understood.

Elements in the 3’ untranslated region (3’ UTR) that govern mRNA stability provide for the regulation of critical regulatory proteins, such as neurotrophic factors [18]. Adenylate- and uridylate (AU)-rich elements (AREs) are 50–150 bp regions in the 3’UTR that serve as binding sites for trans-acting ARE binding proteins (ARE-BPs), which either stabilize or destabilize transcripts [19, 20]. Although the precise consensus sequence of AREs is not well established, AREs are often distinguished by a high AU content and the presence of AUUUA pentamers [19]. Based on current estimates, AREs are predicted to be present in 8% of the human transcriptome. However, only a tiny number of AREs have been experimentally validated as functional targets of the ARE-BPs [20, 21]. TIS11/TTP family ARE-BPs, tristetraprolin (TTP), butyrate response factor 1 (BRF1), and 2 (BRF2) target mRNAs for fast degradation by binding to AREs [22, 23]. On the other hand, numerous studies have shown that epigenetic processes, such as microRNAs (miRNAs), may mediate the relationship between environmental stresses and gene expression [24]. MiRNAs are single-stranded, short (22 bp), noncoding RNAs that can influence gene expression post-transcriptionally [25]. In general, a miRNA acts by binding to the seed sequence in 3’UTR and repressing mRNA production by degradation or translational repression [26].

Several studies have revealed the effect of TTP through the ARE sites on BDNF [27–29], and miRNA-specific databases have estimated that miR-16 has the ability to target BDNF. In this study, we examine the expression of two influential factors, TTP and miR-16, in patients with schizophrenia that can disrupt the regulated expression level of BDNF in a case-control study.

## Materials and methods

### Study design

The selection of the gene and miRNA investigated in this study is based on the design of a regulatory axis in Alzheimer’s disease from our previous research [27]. Briefly, using the AREsite database [30], a database for elements rich in AU and direct evidence for interaction and lifetime regulation, and selecting the ATTTA motif and inserting ENSG00000176697 as the target gene (BDNF), the genes that affect BDNF through the ARE sites were identified. In order to select a miRNA for study, BDNF was input as a target gene into the miRTarBase [31] and miRWalk2.0 [32] databases to identify validated and predicted miRNAs that target the mentioned gene. TargetScan [33] was used to visualize the binding sites of miRNAs to BDNF.

### Participants and samples

This study was carried out within the framework of the Acute Phase Psychiatric Survey (ARAS) [34], recently published in Azerbaijan. The Ethics Committee approved the research protocol of the Medical University of Tabriz (IR.TBZMED.REC.1399.462). Fifty adult patients with recent-onset SCZ (less than two years) and 50 healthy controls of the same age and sex were recruited. The diagnosis was based on the SCID (Structured Clinical Interview for DSM-5 [35]) Diagnostic and Statistical Manual of Mental Disorders in SCZ [36], 5th Edition (DSM-5). Patients with SCZ are drug naïve. In this study, the criteria for exclusion were the 22q11.2 deletion syndrome, intellectual impairment, and substance misuse (with the exception of smoking). To evaluate control participants, the Mini-International Neuropsychiatric Interview [37] was used. Pregnancy, mental problems, or systemic diseases were deemed exclusion criteria for the control group. Individuals with serious mental illness (SMI) in a first-degree family were also excluded. SMI refers to mental diseases that are persistent, need continuing treatment, and have a major effect on function, such as chronic psychotic disorders, bipolar affective disorder, and severe personality disorders [38]. 10 ml of PB was collected after all participants and/or their caregivers provided their written informed consent.

### Quantitative polymerase chain reaction (qPCR)

According to the manufacturer’s protocol, total RNA was extracted from whole blood using the Hybrid-RTM blood RNA purification kit (GeneALL, Seoul, South Korea). Nanodrop was used to evaluate the concentration and quality of extracted RNA (Thermo Scientific, Wilmington, DE). Following the manufacturer’s instructions, cDNA was synthesized using the HyperScript™ kit (GeneAll). The cDNA was prepared and stored at -20 °C for later use. Table 1 lists the primers used to amplify TTP, the housekeeping gene, Hypoxanthine Phosphoribosyltransferase 1 (HPRT1), and unique stem-loop primers for has-miR-16-5p and U6 snRNA as reference RNA. The Step OnePlus™ Real-Time PCR and the RealQ Plus2x Master Mix were used to perform the qPCR (Ampliqon, Odense, Denmark).

**Table 1:** Primers used for the expression assay

List of primers used in this study		
Gene name	Gene reference ID	Primer sequences (5’-3’)
*TTP*	NM_003407.5	Forward primerGACATTCAGAGAAGGGCATCAG Reverse primerAGGCTGCTCAGTAATCCTCTC
*HPRT1*	NM_000194.3	Forward primerAGCCTAAGATGAGAGTTC Reverse primerCACAGAACTAGAACATTGATA
*miR-16-5P*	-	Forward primerAACAGTGTAGCAGCACGTAAA Reverse primerGTCGTATCCAGTGCAGGGT SLP primer GTCGTATCCAGTGCAGGGTCCGAGGTATTCGCACTGGATACGACCGCCAA
*U6*	-	Forward primerGCTTCGGGCAGCACATATCTAAAAT Reverse primerAAAGCCCGAAGCTGTGATGATGC SLP primer GTCGTATCCAGTGCAGGGTCCGAGGTATTCGCACTGGATACGACAAAAATAT

*SLP Stem-loop primer

### Statistical analysis for QPCR

The analysis of the data was carried out with the assistance of the R v.4 software packages brms, stan, pROC, and GGally. We evaluated the expression of hsa-miR-16-5p and TTP in PB samples from healthy controls and SCZ patients. Using the multiple Bayesian quantile regression model, the relative expressions of miR-16-5p and TTP in SCZ patients and healthy controls were compared. We employed the asymmetric Laplace distribution to parameterize the log-transformed dependent variable since relative expressions had a non-normal pattern (relative expressions). We utilized the asymmetric Laplace distribution with location (mu = 0), scale (sigma = 1), and asymmetry parameter quantile (q = 0.5) for quantile regression. Before Sigma, the default brms were taken into consideration (student t). Variables and effects of gender * group interaction with a significant or borderline p-value (*p* < 0.1) in the univariate data analysis were included in the multiple regression model. The final model was gender and age-adjusted. The model with the lowest Pareto smoothed importance-sampling leave-one-out cross-validation (PSIS-LOO) value was chosen [39]. Gender and age effects were adjusted. The adjusted P-values of 0.05 were considered significant. The expression of the aforementioned genes was also evaluated throughout age groups and between males and females. The associations between the research variables were assessed using Spearman correlation coefficients in both SCZ patients and healthy control individuals. The diagnostic power of the genes was determined using a receiver operating characteristic (ROC) curve study. The simulation was used to investigate the effect of n, likelihood, and priors on power. Statistical analyzes were carried out in the R 4.2 environment using the RStan, loo, and brms packages [40].

## Results

### Gene and MiRNA selection

**Fig. 1:**
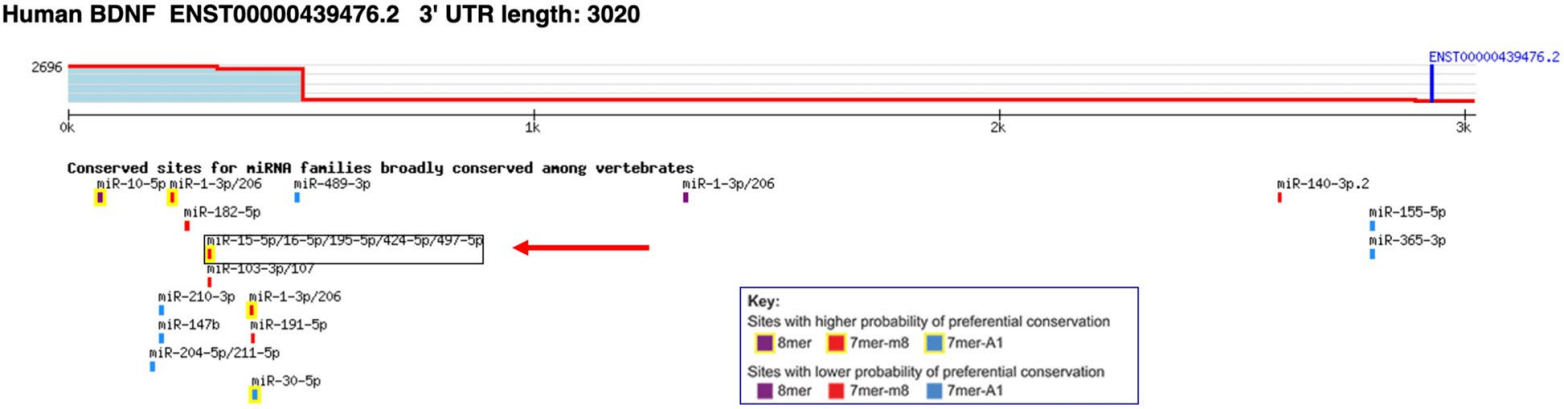
TargetScan’s prediction of the miR-16 binding region on BDNF mRNA.

The AREsite database identified three genes that have the ability to target BDNF gene mRNA and post-transcriptionally regulate its expression, including TTP, HUR1 and AUF1. TTP was selected as the gene of interest, which was introduced in our previous bioinformatics study as one of the significantly up-regulated hub genes (log2FC = 1.236905953, adj.P.Val = 9.06E-07) in SCZ [41]. Among the proposed miRNAs, miR-16 was selected because of its interaction with TTP, which was previously revealed in a study [42], as well as its ability to target the ARE site in addition to the response site to miRNAs [43]. Figure 1 illustrates the binding site of miR-16 on the BDNF mRNA using the TargetScan database.

### General demographic data

We examined 50 patients (male/female: 22/28) with an age (mean ± standard deviation (SD)) of 35.9 ± 5.6 and 50 healthy controls (male/female: 23/27) with an age (mean ± SD) of 34.7 ± 5.4 who were of Turkish Azeri ethnicity.

### QPCR data analysis

**Fig. 2:**
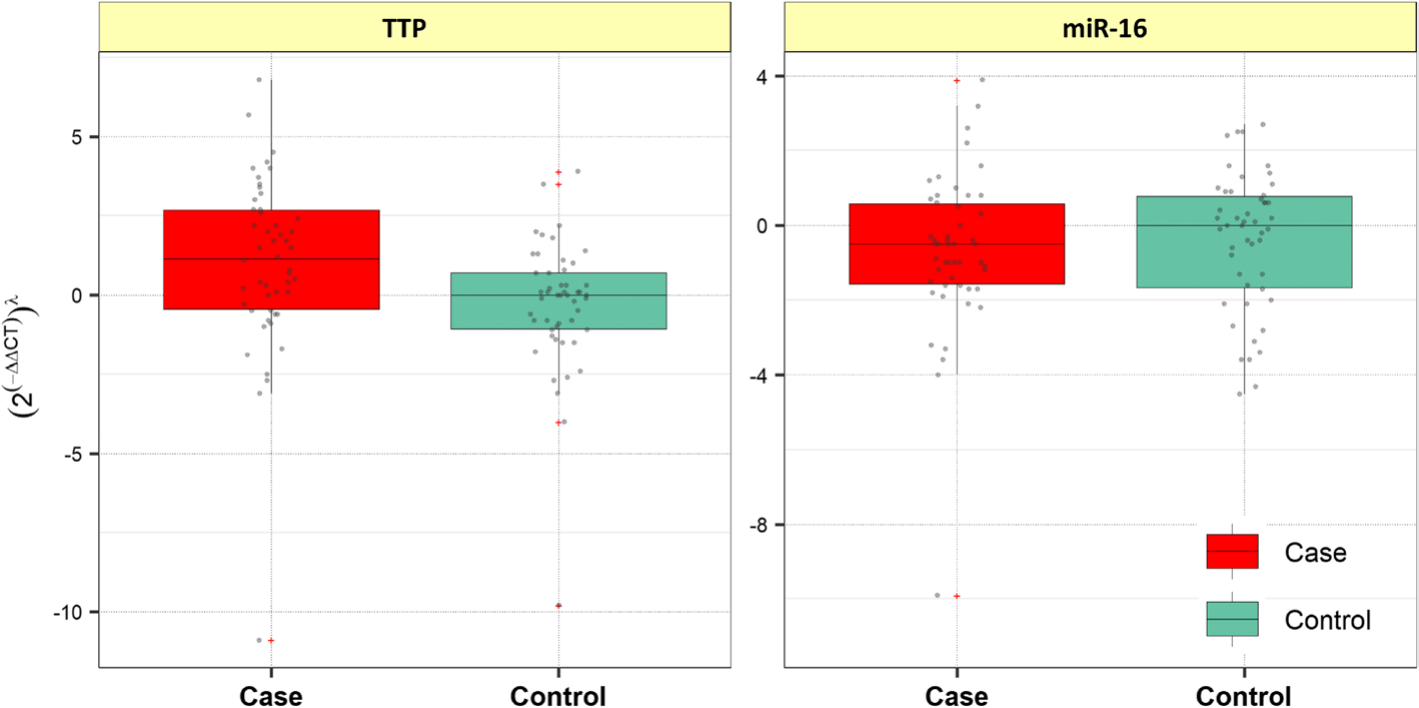
Expression of hsa-miR-16-5p and TTP in peripheral blood samples from cases and controls’ peripheral blood samples. Values are represented as gray dots. The means of the expression levels and the interquartile range are shown

Figure 2 shows the relative expression levels of the TTP gene and miR-16 in patients and controls. TTP expression levels were higher in patients than controls, regardless of participants’ gender and age (posterior beta = 1.532, adjusted *P*-value = 0.012). When the case and control groups are not considered and the expression of TTP is examined in the male and female groups of participants, no significant change in TTP levels is observed. Considering the gender of study participants (male cases vs. male controls, and female cases vs. female controls), the analysis showed that the *TTP* expression levels were significantly higher only in female subjects compared to female controls (posterior beta = 1.685, adjusted *P*-value = 0.001). MiR-16 expression levels did not show significant differences in PB samples between SCZ patients and healthy controls (adjusted *P-value* = 0.248). Tables 2 and 3 provide detailed information on the relative expression of TTP and miR-16, respectively.

**Table 2:** The association between Schizophrenia and TTP relative gene expression: The results of the Bayesian quantile regression model

	TTP	Posterior Beta of (2^(−ddct)^)^ʎ^	SE	Adjusted *P*-value*	95% Crl for Beta
Total	Group, Case vs. control	**1.532**	**0.37**	**0.012**	**[0.84, 2.25]**
	Sex, Male vs. Female	0.795	0.43	0.122	[-0.07, 1.63]
	Age (years)	-0.041	0.02	0.089	[-0.09, 0.01]
	Group * Sex	-1.375	0.54	0.02	[-2.43, -0.34]
Male	Case vs. control	-0.011	0.37	0.741	[-0.67, 0.75]
	Age	-0.017	0.03	0.93	[-0.08, 0.04]
Female	Case vs. control	**1.685**	**0.38**	**0.001**	**[0.94, 2.42]**
	Age	-0.059	0.04	0.087	[-0.13, 0.01]

*Estimated from frequentist methods; *CrI* Credible interval, ʎ: Power transformation value estimated from Box-cox or Yeo-Johnson methods

**Table 3:** The association between Schizophrenia and mir16 relative gene expression: The results of the Bayesian Quantile regression model

	miR-16	Posterior Beta of (2^(−ddct)^)^ʎ^	SE	Adjusted *P*-Value*	95% Crl for Beta
Total	Group, Case vs. control	-0.469	0.31	0.248	[-1.04, 0.15]
	Sex, Male vs. Female	-0.241	0.36	0.842	[-0.95, 0.46]
	Age (years)	0	0.02	0.814	[-0.04, 0.04]
	Group * Sex	0.171	0.49	0.77	[-0.81, 1.11]
Male	Case vs. control	-0.233	0.3	0.534	[-0.81, 0.36]
	Age	0.053	0.03	0.311	[0.002, 0.1]
Female	Case vs. control	-0.433	0.32	0.362	[-1.06, 0.2]
	Age	-0.038	0.03	0.417	[-0.09, 0.02]

*Estimated from frequentist methods; *CrI* Credible interval, ʎ: Power transformation value estimated from Box-cox or Yeo-Johnson methods

### Correlation analysis

**Fig. 3:**
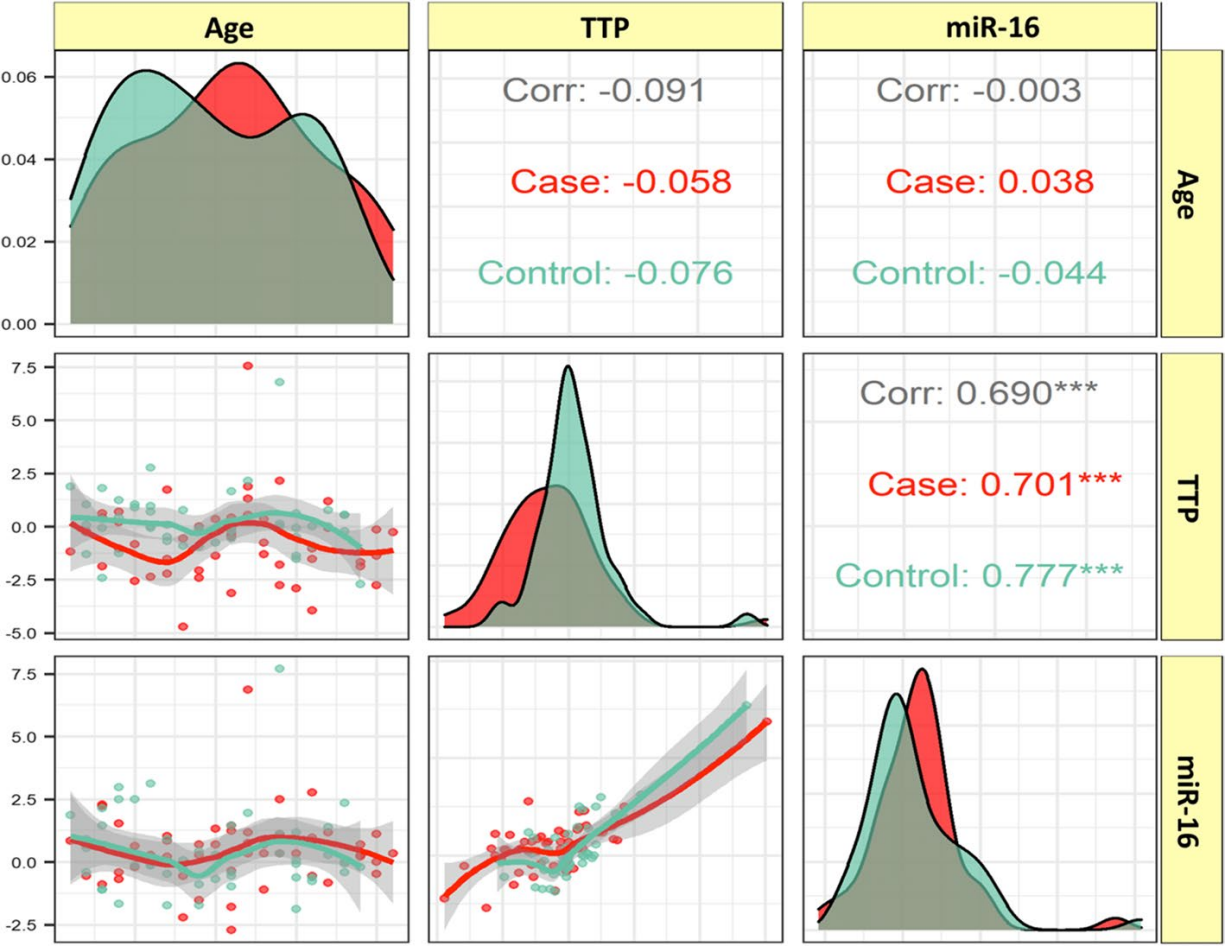
Correlations between the expression levels of the TTP and miR-16 genes and age. The variable distribution is depicted on the diagonal. Bivariate scatter plots with a fitted line are shown in the lower portion of the diagonal. Correlation coefficients and P-values are shown in the upper part of the diagonal

TTP and miR-16 expression were not correlated with participants’ age. The expressed levels of the TTP and miR-16 were significantly correlated both among SCZ patients and among healthy controls (*r* = 0.701, *P* < 0.001 and *r* = 0.777, *P* < 0.001, respectively) (Fig. 3).

### ROC curve analysis

**Fig. 4:**
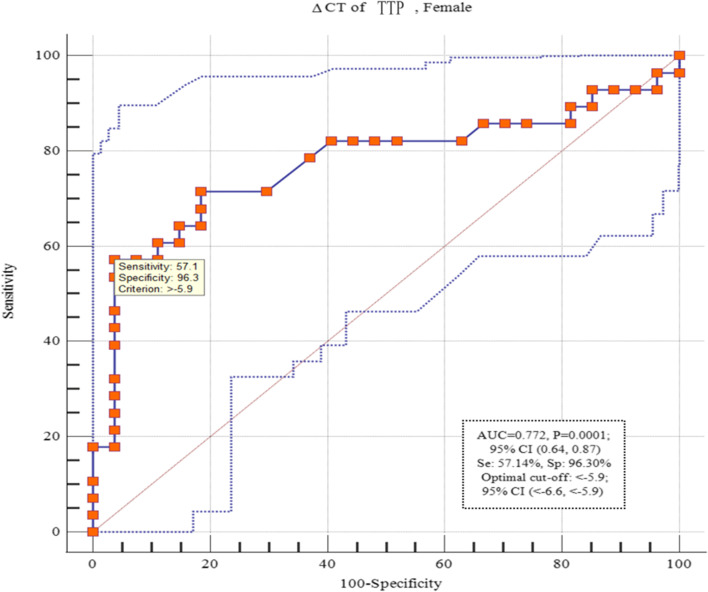
ROC curve analysis. The TTP transcript level showed a diagnostic power of 0.772

We assessed the diagnostic power of TTP to distinguish female SCZ patients from healthy controls at different threshold settings. We classified them as candidates for biomarkers according to the decreasing area under the receiver operating characteristic (ROC) curve (AUC) and graphed the results. TTP transcript level presented a diagnostic power of 0.772 (95% confidence interval, 0.64–0.87; *P* = 0.0001) with a specificity of 96.3% and a sensitivity of 57.1% (Fig. 4), based on the area under the ROC curves.

## Discussion

In this study, we tried to investigate the importance of two factors, TTP and miR-16, by examining their expression, which affects the expression of BDNF in the peripheral blood of patients with SCZ but not in brain tissue. BDNF expression has not been assessed in this study; instead, many studies have shown altered expression of this neurotrophin in the brains of people with SCZ. In this regard, some researchers have focused on brain BDNF levels in postmortem examinations of SCZ patients’ postmortem examinations. Durany et al. revealed a considerable rise in BDNF concentrations in cortical regions and a significant drop of this neurotrophin in patients’ hippocampus compared to controls [44]. Similarly, Takahashi et al. discovered that brain-derived neurotrophic factor (BDNF) levels were explicitly enhanced in the anterior cingulate cortex and hippocampus of SCZ patients, but TrkB receptor expression was dramatically decreased in the hippocampus and prefrontal cortex [45]. BDNF mRNA and protein levels in the dorsolateral prefrontal cortex (DLPFC) of SCZ patients were found to be significantly lower than in normal persons. BDNF mRNA and protein levels in the DLPFC of SCZ patients were found to be significantly lower than in normal individuals [46]. These results are significant because they provide more support for the neurotrophin theory of SCZ psychoses. This technique, however, restricts the potential for conducting a dynamic analysis of variations in BDNF levels in connection to the SCZ process and its clinical progression.

Measurement of plasma or serum levels is a reliable method of evaluating BDNF involvement in SCZ patients [47]. The potential of BDNF to permeate the blood-brain barrier means that BDNF levels measured in the peripheral blood may represent levels in the brain [46]. In this context, an analysis identified a connection between peripheral and brain BDNF, with simultaneous variations in BDNF levels in plasma and CSF in SCZ-positive individuals, revealing that plasma BDNF levels mirror brain BDNF levels [48]. In trials with treatment-naive individuals who have experienced their first psychotic episode, plasma levels of BDNF were shown to be significantly lower (135 ± 21.77 pg/ml) compared to control participants (290.5 ± 38.8 pg/ml) [49]. Another research in a comparable group discovered that BDNF levels in serum were considerably lower in individuals with first-episode SCZ (23.92 ± 5.99 ng/ml) compared to control people (30, 0 ± 8.43) [50]. Lower significant BDNF levels were identified in first-episode SCZ patients than in healthy control participants (9.0 ± 4.2 ng/ml vs. 12.1 ± 2.2 ng/ml), and a significant positive connection between BDNF levels and the PANSS positive subscore was reported [51]. On the other hand, in 2006, one of the first studies that prospectively analyzed the development of BDNF plasma levels in patients with a final diagnosis of SCZ measured them at baseline at the commencement of their first psychotic episode (4.19 ± 2.26 ng/ml) and the following months after commencing treatment (typically with atypical antipsychotics), with a greater level at six months duration (6.53 ± 2.48 ng/ml), nearing the level of control subjects (7.55 ± 4.31 ng/ml) [52]. The high number of studies that indicated decreased levels of BDNF in the brain tissue and blood of patients with SCZ prompted us not to investigate the BDNF levels in the present study.

In addition to identifying TTP in the AREsites database [53] as a factor that has the potential to target BDNF, in 2014, Kumar et al. mentioned TTP as the novel regulator of BDNF in their results [28]. This work showed that ARE-BPs TTP and its family members Butyrate Response Factor 1 (BRF1) and 2 (BRF2) adversely affect the production of BDNF-S and BDNF-L containing transcripts in numerous cell lines and that the interaction between TTP and the AU-rich region at the proximal 5’ end of the BDNF 3’ UTR is direct. Endogenous BDNF mRNA co-immunoprecipitates with endogenous TTP in differentiated mouse myoblast C2C12 cells, and TTP overexpression destabilizes BDNF-S-containing transcripts. Finally, RNAi-mediated elimination of TTP improves the amount of endogenous BDNF protein in C2C12 cells [28]. However, the identification of BDNF as the target of miR-16 by two databases, miRTarBase [31] and miRWalk2.0 [32], completed another part of our study thesis. In a study conducted in the hippocampus of rats modeled after depression induced by maternal deprivation (MD) and chronic unpredictable stress, an increase in miR-16 expression was observed, followed by a down-regulation of BDNF [54]. In another study on a rats model, miR-16 overexpression decreased BDNF expression and indicated that miR-16 contributes to the regulation of apoptosis and autophagy and could account for some part of the therapeutic effect of selective serotonin reuptake inhibitors [55].

Several lines of evidence show that whole-genome expression investigations may help identify molecular changes in mental illnesses. In this regard, an overlap has been observed in gene expression profiles in blood cells and postmortem brains, supporting the hypothesis that studies in peripheral tissues may provide new information on SCZ pathogenesis and innovative biomarkers for diagnostic evaluation and personalized treatment [56–58]. Post-transcriptional regulation by numerous factors can be concluded; RBPs and miRs are among the most fundamental of these factors [59], with the slightest alteration in the regulatory network imposed by RBPs and miRs leading to a larger scale change in disease manifestation [60]. In this study, we investigated the expression of TTP and miR-16 in SCZ patients in a case-control study with a sample size of 50 patients with SCZ and 50 healthy controls. TTP expression levels in patients were higher than in controls, regardless of gender or age (posterior beta = 1.532, adjusted *P*-value = 0.012). When the gender of study participants was considered, the analysis revealed that TTP expression levels were significantly higher only in female subjects (posterior beta = 1.685, adjusted *P*-value = 0.001). There were no significant differences in miR-16 expression levels in PB samples between SCZ patients and healthy controls (adjusted *P*-value = 0.248). The high expression of TTP in female subjects may be rooted in SCZ characteristics. The symptoms of SCZ differ significantly based on gender. Men with SCZ have worse negative symptoms and clinical manifestations than women. Men exhibit more severe symptoms of social withdrawal and drug use, whereas women frequently exhibit agitated, depressive, and emotional symptoms [61]. Gender-related factors such as sex gland hormones and sex chromosomes could account for these differences [62].

**Fig. 5:**
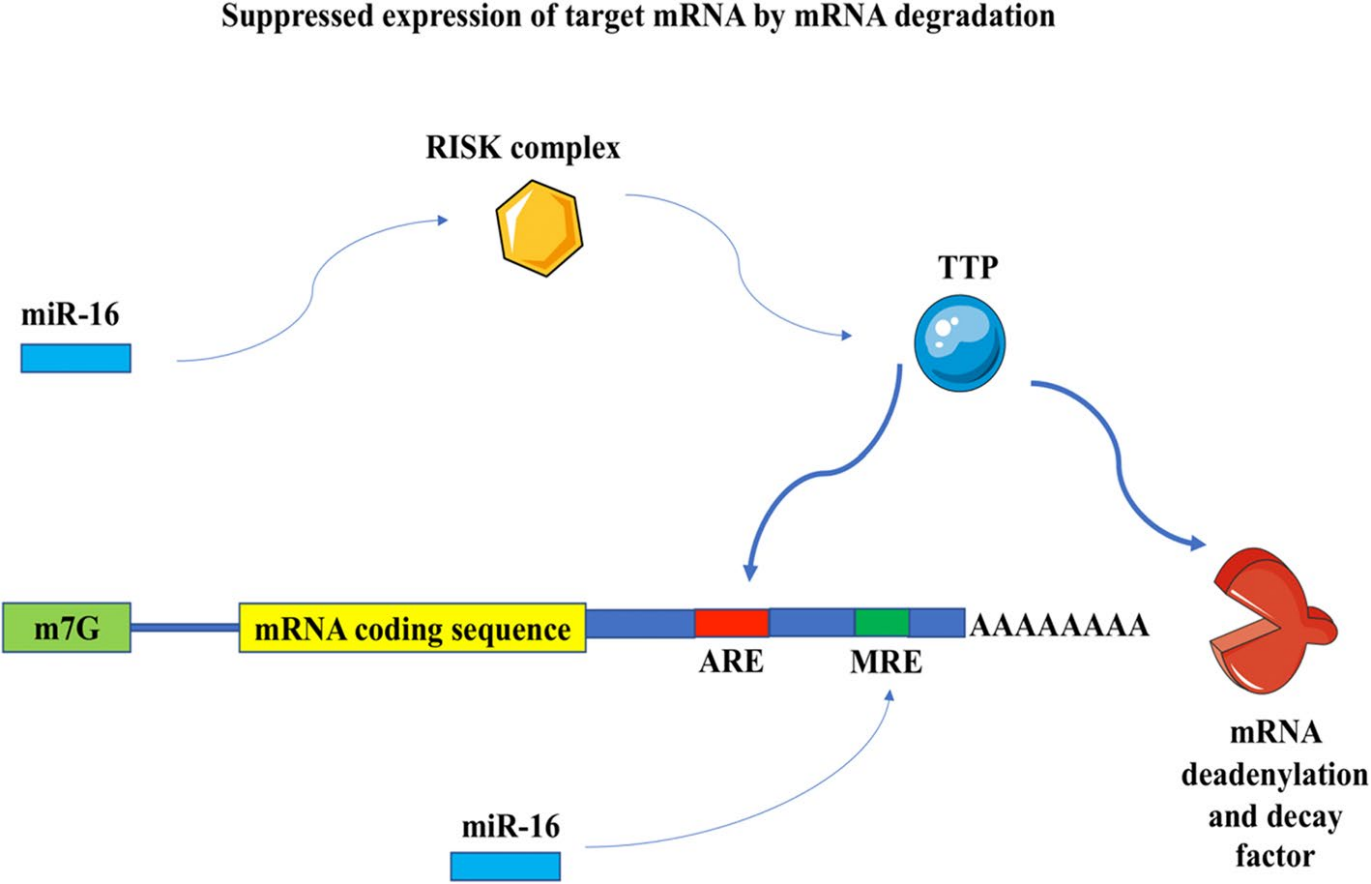
Schematic representation of the mechanism of action of TTP in collaboration with miR-16 in effect on ARE. TTP is an RBP that regulates the expression of target mRNAs post-transcriptionally by acting on ARE and recruiting deadenylation and degradation factors. TTP is assisted in this operation by miR-16 in the form of a RISC complex. MiR-16 can also affect gene expression through microRNA response elements (MRE).

It should be noted that the miR-16 sequence contains a UAAAUAUU sequence that complements the ARE site, which was one of the reasons for choosing it among other miRs. There is an indirect collaboration between TTP and miR-16 via members of the RISK complex, and TTP may help miR-16 target ARE-included mRNAs (Fig 5) [42]. Based on the results of our correlation analysis, the presence of positively correlated gene expression between TTP and miR-16 could imply the same. In addition, one of the targets of miR-16 is the serotonin transporter (SERT), which causes serotonin reuptake and is the pharmacological target of selective serotonin reuptake inhibitor (SSRI) antidepressants. SERT expression levels are reduced when miR-16 levels are elevated due to long-term fluoxetine treatment [63]. The positive effect of SSRIs on the recovery process of SCZ [64] can be achieved by targeting SERT and reducing expression by increasing miR-16 expression due to the role of SERT in SCZ [65].

It should be noted that a direct study of the effect of TTP and miR-16 on BDNF through the peripheral blood of patients with SCZ is probably not possible, this is one of the limitations of our study and requires histological or cell line studies. However, this effect on BDNF in animal models and cell line studies has been proven in cases other than SCZ. Based on ROC curve analysis with a power of 0.772, TTP has the potential to act as a diagnostic indicator in this disease. On the other hand, miR-16 is one of the main determinants in the drug response to SSRIs, and although its expression in the blood of people with SCZ was not significantly different from controls, it does not mean that this condition is the same in the brain and the central nervous system and needs further studies.

For future research, it is suggested that the precise relationship between miR-16 and TTP be investigated in cell culture and animal studies, as well as its function on its target mRNAs, which in this study correlate with SCZ. In this study, we rely on the results of many previous studies on BDNF levels. Subsequent studies should also measure BDNF levels to determine the exact relationship between these two factors and BDNF levels.

## Conclusions

In conclusion, the current study is the first evidence to highlight the expression of the TTP gene and miR-16 in the periphery of SCZ patients. Our findings could shed light on the pathogenesis of SCZ concerning BDNF levels. Further research with larger sample sizes and paired PB and OE samples from drug-free cases can significantly strengthen these findings.

## Data Availability

The datasets used and/or analyzed during the current study are available from the corresponding author on reasonable request.
